# Case report of three patients with end-stage recurrent glioblastoma treated with meldonium

**DOI:** 10.1038/s44276-025-00124-7

**Published:** 2025-04-28

**Authors:** Sandra Bien-Möller, Martin E. Weidemeier, Josefine Radke, Jörg Baldauf, Stefan Engeli, Mladen V. Tzvetkov, Henry W. S. Schroeder

**Affiliations:** 1 Department of General Pharmacology, University Medicine Greifswald, Greifswald, Germany; 2 Department of Neurosurgery, University Medicine Greifswald, Greifswald, Germany; 3 Institute of Pathology, University Medicine Greifswald, Greifswald, Germany; 4 Department of Clinical Pharmacology, University Medicine Greifswald, Greifswald, Germany

## Abstract

**Background:**

Glioblastoma is the most aggressive primary brain tumor in adults. The prognosis is still very poor with a median survival time less than a year. A growing body of data supports the role for fatty acid oxidation (FAO) in the aggressive behavior of glioblastoma. We have previously shown that meldonium, an orally active compound that impairs FAO, caused significant growth reduction of glioblastoma in mice. Here, we report three cases of experimental meldonium-containing therapy in end-stage recurrent glioblastoma patients.

**Methods:**

Three end-stage glioblastoma patients, who had second relapse tumor progression after standard of care therapy, received 500 mg meldonium twice a day on the top of the existing therapy regimen. Tolerability and treatment outcomes were monitored.

**Results:**

Meldonium was well tolerated by all three patients. One patient experienced long-term growth arrest and maintained clinically stable disease status, currently 24 months into treatment with meldonium. In contrast, the other two patients passed away.

**Conclusions:**

The case reports presented here suggest good tolerability and the potential for meldonium to improve outcome in glioblastoma patients. Controlled clinical trials need to follow to evaluate systematically possible benefits from the integration of meldonium into standard glioblastoma treatment protocols.

## Introduction

Glioblastoma (GBM) is the most prevalent, highly aggressive malignant brain tumor in adults. GBM accounts for 50.1% of all malignant brain tumors in the United States and has a very poor prognosis with a 5-year survival rate of 6.9% [1]. First-line treatment for GBM is surgery, followed by adjuvant radiotherapy and temozolomide application according to Stupp-protocol [2]. Nevertheless, response rates are still very poor and postoperative recurrence occurs in about 90% of GBM patients [3]. So far, standard treatments for recurrent GBM are not well defined. Targeted therapies and immunotherapies have had limited success in GBM so far [4]. GBM cells reprogram their metabolism to promote cell survival and invasion. New evidence suggests that this metabolic reprogramming also mediates resistance to the standard GBM therapies [5].

Fatty acid oxidation (FAO) seems to be responsible for supplying the tumor with substantial additional energy. The importance of a functional fatty acid metabolism for proliferation and for in vivo tumor growth already had been demonstrated [6, 7]. Depending on the intra-tumoral conditions, FAO can promote GBM progression, acts as an alternative energy source and appears to be involved in the metabolic plasticity of GBM to adapt to the dynamic nutrient microenvironment [8, 9]. This makes FAO, along with glycolysis [10] a promising therapeutic target.

One of the most important factors that enables FAO to function properly is L-carnitine as it is an indispensable co-factor for transport of activated fatty acids into mitochondria where FAO takes place. The most relevant and high affinity L-carnitine uptake transporter is OCTN2 (SLC22A5) [11]. OCTN2 is mainly detectable in the intestine, kidney, placenta and heart muscle. Its expression in the brain particularly seems to be worth mentioning, especially as a component of glial cells and the blood-brain barrier [11]. Our group demonstrated that expression of OCTN2 is increased in GBM compared to healthy brain tissue [12] implying a role in GBM progression which could possibly be attributed to the GBM’s dependence on a functioning FAO. In addition, we showed a poorer prognosis for GBM patients with a high expression of OCTN2 [12]. In vitro, L-carnitine supplementation increased GBM cell viability while siRNA-mediated OCTN2 silencing resulted in loss. Most importantly, treatment of mice with the OCTN2 inhibitor meldonium leads to reduced tumor growth in an orthotopic GBM mouse model [12]. Meldonium is a well-established drug in Eastern Europe (Mildronate® from Grindeks) for treatment of central nervous and cardiac ischemic diseases [13]. Meldonium is not approved in the European Union or US and is known here exclusively as doping agent. In addition to the inhibition of OCTN2, meldonium inhibits the L-carnitine synthesis from its biochemical precursor, γ-butyrobetaine, and blocks the mitochondrial carnitine palmitoyl-transferases (particularly CPT1) and thus FAO [13, 14]. In view of this multitude interaction with the carnitine-dependent cell metabolism, the promising results from in vitro and in vivo studies [12, 15, 16], and given the existing drug status in some countries, meldonium seems quite conceivable as a potential add-on therapy for GBM.

We report here on three patients with recurrent GBM who received meldonium in addition to their existing therapeutic regimens as part of an experimental therapy due to the unavailability of suitable alternative treatment.

## Methods

The report presents an experimental therapeutic use of meldonium. All three patients signed a consent form which included their acknowledgment of having been informed about the general conditions of the experimental therapy, the nature of this particular medicinal product (meldonium), and their wish to participate in the experimental study. An appropriate institutional review board approved the project.

Patients with a second relapse, who were not eligible for further surgical intervention, were treated with meldonium on top of their existing therapeutic regimes. They were taking 500 mg meldonium twice daily. The intake of meldonium was planned as repeated 3-month cycles, with a break of 2 weeks between each treatment cycle.

Tumor size was determined using volumetric analysis on follow-up MRI scans, focusing on T1 contrast-enhancing lesions and T2-weighted Fluid attenuated inversion recovery (FLAIR) hyperintensities. Patients rated their tolerability of 26 distinct symptoms on a scale from 0 (absent) to 5 (extremely intense). Items of the short form 36 (SF-36) questionnaire was utilized to self-assess Quality of Life.

## Results

Three patients underwent experimental treatment with meldonium. The patient characteristics are given in Table 1.

**Table 1:** Summary of important patient data (RTx, radiotherapy; PRGBM, primary GBM; TMZ, Temozolomide; TTF, Tumor Treating Fields; *until the latest MR imaging in July 2024).

	PATIENT #1	PATIENT #2	PATIENT #3
AGE AT DIAGNOSIS	66	62	64
SEX	male	male	male
NEUROPATHOLOGICAL PARAMETERS			
*MGMT PROMOTER*	methylated	non-methylated	non-methylated
*IDH1*	wildtype	wildtype	wildtype
THERAPIES CARRIED OUT			
*PRIMARY GBM*	TMZ + RTx (Stupp regimen) TTF	TMZ + RTx (Stupp regimen)	TMZ + RTx (Stupp regimen) TTF
*1ST RELAPSE*	RTx TMZ TTF	Avelumab anti-VEGFR vaccine CCNU/VP16 RTx TMZ	CCNU TTF
*2ND RELAPSE*	TMZ TTF	TMZ	Dabrafenib Trametinib
*3RD RELAPSE*			CCNU Avastin
START OF MELDONIUM INTAKE	after 2nd relapse	after 2nd relapse	1.) after 2nd relapse for 19 days 2.) after 3rd relapse for 6 weeks
DURATION OF MELDONIUM INTAKE	still ongoing	about 2 months	1.) 19 days (followed by a break of about 4.5 months) 2.) 6 weeks
SURVIVAL STATUS	alive	deceased	deceased
OVERALL SURVIVAL TIME SINCE PRIMARY DIAGNOSIS	*1969 days (64.6 months, 5.4 years)	655 days (21.5 months, 1.8 years)	713 days (23.4 months, 2.0 years)
SURVIVAL TIME AFTER MELDONIUM	*749 days (24.6 months, >2.0 years)	68 days (2.3 months)	199 days (6.6 months) after initiation; 43 days (1.4 months) after resumption

### Case 1

**Fig. 1:**
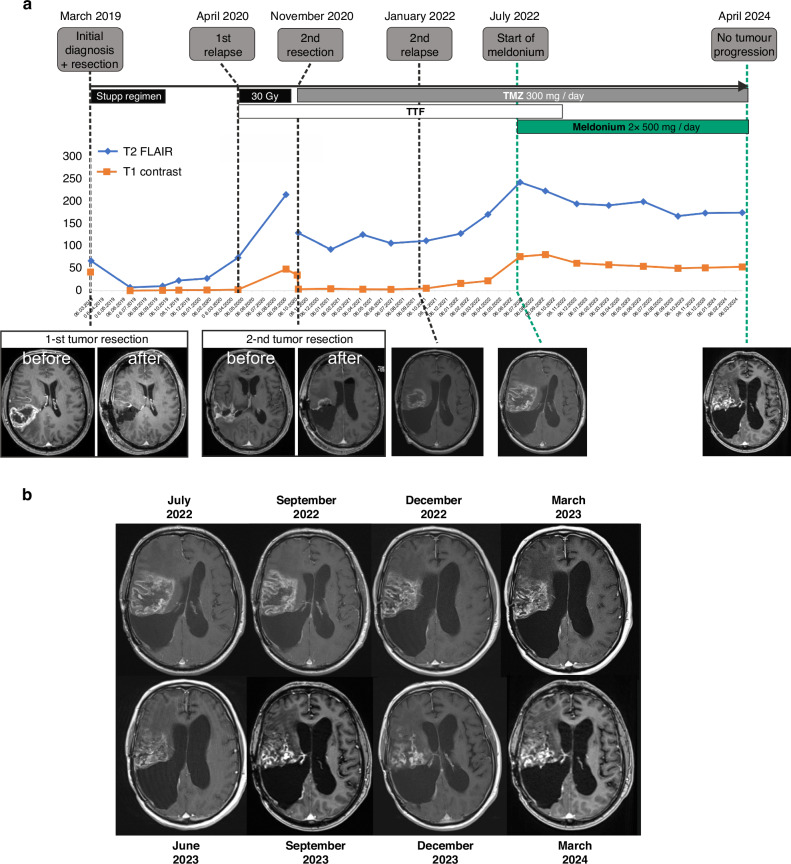
Response of patient #1 to GBM therapy before and after the addition of meldonium. **a** Schematic representation of the clinical course of the disease, including surgical interventions and radiochemotherapies. Tumor volumetry for FLAIR and T1-contrast weighted MRI after gadolinium injection are also shown, along with original MRI images to illustrate surgical resections and tumor relapses. **b** A detailed representation of representative MRI images available under meldonium treatment, illustrating the lack of tumor progression under this condition. TTF Tumor Treating Fields therapy, TMZ temozolomide; 30Gy radiotherapy totaling 30 Grays.

The first patient (#1), a 66-year-old male, was diagnosed with right parietal lobe primary Isocitrate-Dehydrogenase (IDH) wildtype glioblastoma in March 2019. Surgical intervention led to a gross total resection, confirmed by postoperative MRI and neuropathological evaluation indicating a methylated MGMT promoter status. The patient’s postoperative regimen included adjuvant radiation therapy (60 Gy) starting in May 2019 and six cycles of adjuvant temozolomide treatment from June to November 2019 (Stupp regimen [2]), alongside initiation of Tumor Treating Fields (TTF) therapy in June 2019. Despite these interventions, MRI in January 2020 unveiled multifocal tumor recurrence, with subsequent MRIs indicating continued tumor growth (Fig. 1a).

Following the first relapse, the patient underwent additional radiotherapy (30 Gy) in June 2020. However, the tumor’s continued progression necessitated a second surgical resection in October 2020. The second resection was only partial due to the tumor’s infiltration into the corticospinal tract. Post-second resection, the patient resumed temozolomide (300 mg) and continued with TTF therapy. A second tumor recurrence was confirmed by MRI in January 2022, with ongoing treatment failing to halt tumor progression (Fig. 1a).

This led to the patient’s decision to commence experimental treatment with meldonium in July 2022, which he tolerated well. Over the course of meldonium treatment, MRIs conducted at regular three-month intervals demonstrated no significant tumor progression, with even slight reductions in lesion size and FLAIR signal observed, marking a stable disease state (Fig. 1b).

The patient reported a decline in well-being during the second break in the meldonium intake after the second 3-month therapy cycle. The decline in the well-being improved upon resuming the medication, leading to a continuous intake since and exclusion of further breaks. The latest follow-up in March 2024 showed continued disease stability (Fig. 1b). The survival time is 64.6 months (5.4 years). The progression-free survival, starting with the initiation of the meldonium intake, is 24.6 months (>2.0 years). The patient’s ongoing treatment includes meldonium and temozolomide.

**Fig. 2:**
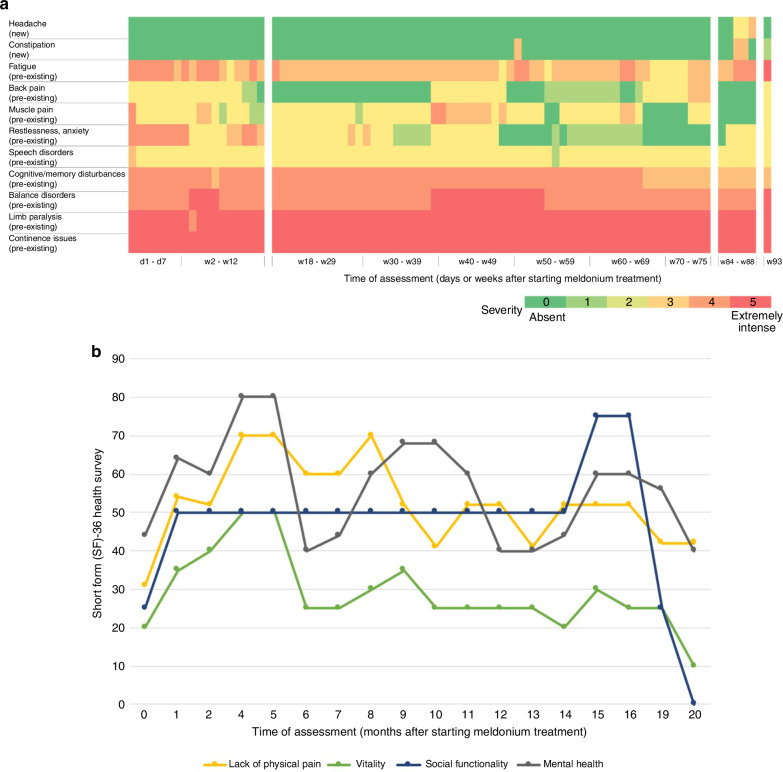
Evaluation of the side effects in patient #1. **a** Lineplot illustrating SF-36 results in patient #1 during meldonium administration. **a** Heatmap illustrating the symptoms observed. A daily monitoring of symptoms was performed during the initial week of treatment (d1-d7), followed by a weekly assessment thereafter (w2-w93). A data collection hiatus occurred between weeks 13 and 17, weeks 76 and 83, and weeks 89 and 92. Notably, constipation and headache emerged as new symptoms after the commencement of meldonium intake, while the other symptoms were pre-existing. d, day; w, week. **b** Quality of life was evaluated using the short form 36 health survey (SF-36). The evaluation was conducted monthly over a period of 20 months. Only complete datasets are displayed due to missing data, a data collection hiatus occurred between months 17 and 18. Notably, an initial improvement in all items was observed during the first 6 months of administration. A comparison to the initial assessment reveals a stable condition with a slight improvement over time for the first 16 months. Lately a decline has been noted predominantly in vitality and social functionality.

Side effects of meldonium were predominantly mild and consisted mainly of pre-existing conditions, with constipation as the only new mild symptom (Fig. 2a). Quality of life assessments revealed notable improvements in physical functioning and mental health in the first 16 months of meldonium treatment, while social and vitality scores remained stable (Fig. 2b). However, lately some clinical deteriorations were observed (Fig. 2), mainly attributed to long-standing disease and multimodal therapies in light of an aging patient.

### Cases 2 and 3

In contrast to the very successful case reported above, the next two patients died due to the disease despite meldonium intake.

The second patient (#2), diagnosed with right temporobasal glioblastoma in December 2019, underwent a gross total resection at our university hospital. Postoperative assessments confirmed IDH wildtype glioblastoma, with unmethylated MGMT promoter. Following surgery, the patient received radiochemotherapy (Stupp regimen). A recurrence was noted (first relapse) in July 2020. At that point, the patients refused second surgery, continued temozolomide therapy, and participated in the VXM01-AVE-04-INT trial from December 2020 to January 2021. Within this trial, he received avelumab (a PD-L1 antagonist) and an oral anti-VEGF vaccine. However, due to disease progression, he discontinued his participation in the trial. Subsequent treatments included Volumetric Intensity Modulated Arc Therapy and second-line therapy with lomustine and etoposide, both of which were followed by tumor progression. A second navigation-assisted subtotal tumor resection was performed in June 2021 but was quickly followed by disease progression.

**Fig. 3:**
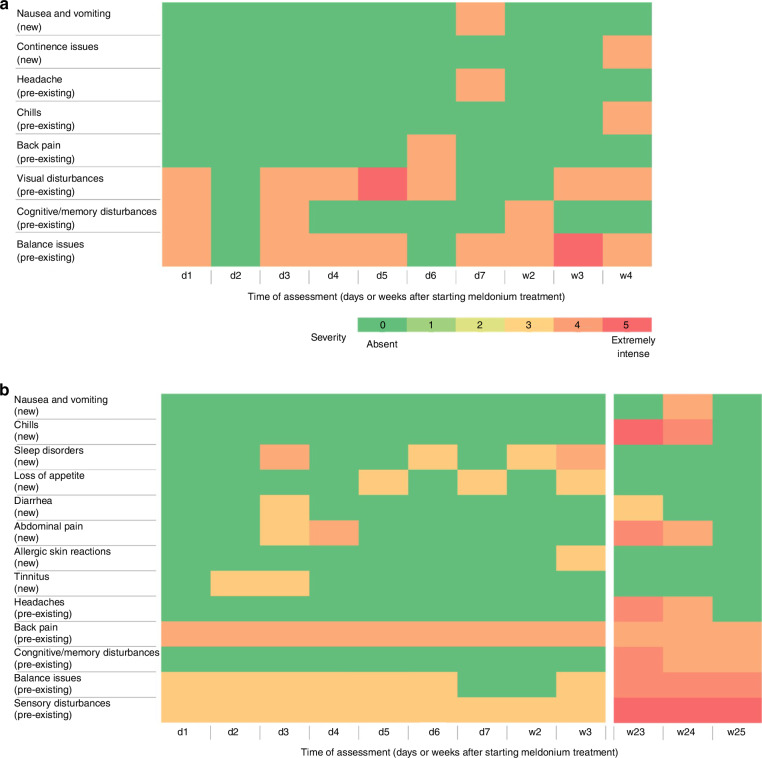
Heatmap illustrating the symptoms observed in patient #2 and #3 during meldonium administration. **a** Heatmap of patient #2: a daily monitoring of symptoms was performed during the initial week of treatment (d1-d7), followed by a weekly assessment thereafter (w2-w4). Notably, nausea and vomiting, along with incontinence issues, emerged as new symptoms post the commencement of meldonium intake, while the other symptoms were pre-existing. **b** Heatmap illustrating the symptoms observed in patient #3 during meldonium administration. A daily monitoring of symptoms was conducted during the initial week of treatment (d1-d7), followed by a weekly assessment thereafter (w2-w25). A data collection hiatus occurred between weeks 4 and 22 due to the discontinuation of meldonium intake, attributed to the patient’s lack of confidence in the medication’s efficacy. Notably, nausea and vomiting, chills, sleep disorders, loss of appetite, diarrhea, abdominal pain, allergic skin reactions, and tinnitus emerged as new symptoms post the commencement of meldonium intake, while the other symptoms were pre-existing. d day; w week.

In August 2021, the patient began experimental therapy with meldonium in a highly progressive tumor state (Supplementary Fig. 1), showing good tolerability. Ultimately, patient #2 died due to the disease in October 2021, 655 days (21.5 months, 1.8 years) post-diagnosis and 68 days after initiating meldonium treatment. Side effects of meldonium were minimal, with no new severe symptoms reported (Fig. 3a). Further details of the disease course are provided in the supplementary data.

The third patient (#3) did not receive his standard GBM treatment in our clinic. He contacted us asking specifically for experimental meldonium treatment. The patient #3 was a 64-year-old male, who was diagnosed with right temporal IDH wildtype glioblastoma with an unmethylated MGMT promoter in March 2021. A gross total resection was performed in April 2021, with subsequent temozolomide and radiotherapy per the Stupp regimen. Maintenance therapy and TTF began in July and August 2021, respectively. After a first relapse, a second gross total resection surgery was performed in December 2021. Second-line therapy with lomustine was initiated, and TTF was continued. Following a second relapse in March 2022, a third gross total resection surgery was performed. A targeted therapy with dabrafenib and trametinib was started.

The patient joined the experimental meldonium therapy in September 2022 but discontinued it after three weeks due to loss of confidence in the treatment. Despite stable MRI scans initially, a third relapse in December 2022 led to a fourth surgical intervention, leading to subtotal resection and adjuvant treatment with lomustine and avastin. He resumed meldonium together with lomustine treatment in February 2023 but again discontinued meldonium intake six weeks later. The patient #3 passed away 713 days (23.4 months) post-diagnosis and 14 days after discontinuing the second period of meldonium treatment.

Side effects of patient #3 during meldonium treatment included new and escalating symptoms, notably nausea, vomiting, and abdominal pain, with a moderate to high severity noted in the third and fourth week (Fig. 3b). No data about quality of life was available. Further details of the disease course are provided in the supplementary data.

## Discussion

Building up on our preclinical data, which showed an impact of OCTN2 expression on GBM patient survival and a significant growth-reduction using the OCTN2 inhibitor meldonium in an orthotopic GBM mouse model [12], we report here the experimental use of meldonium in patients with recurrent end-stage GBM. The present paper reports three cases of recurrent end-stage GBM patients who were taking meldonium in addition to their existing treatment regimens. Meldonium was well tolerated by all three patients. However, the outcomes were very different. In one patient (#1) a longer-term growth arrest of more than 24 months has been achieved so far. This patient went from rapid tumor progression in growth arrest when meldonium was added to the therapy and after all other previous therapies have failed. For patient #1, the very first MRI was performed three days after starting meldonium administration, showing a massive tumor progression compared to the images taken three months earlier. The growth arrest and the subsequent growth reduction were observed three months after the start of meldonium administration. Currently, case #1 presents the classical signs of response and stable disease in MRI according to RANO, also including non-enhancing T2/FLAIR. Thus, an association between the onset of the meldonium effect and the growth arrest could be assumed.

The use of meldonium does not cause any serious side effects. All patients tolerated the intake of the substance well. The physical constraints observed were already present before taking meldonium and were not exacerbated by it. The lack of severe side effects is well described for meldonium [13]. These facts and our patient data regarding the good tolerability of meldonium imply a safe use in patients suffering from GBM. This confirms the data from our animal model, which also showed no side effects that could be attributed to meldonium treatment [12].

This represents a translation of the available in vitro and mice data [6, 9, 12, 15] in humans. Moreover, our report demonstrates that meldonium may be used not only for treating stereotactically induced glioblastoma in a mouse model [12], but have the potential to improve therapy in humans. Similarly to humans, we also observed some heterogeneity in the response to meldonium in the mouse study. The effects of meldonium in mice ranged from strong tumor reduction and growth stagnation to a very limited increased tumor growth only [12]. It may be that only the very good response in mice with strong tumor reduction is translatable to a stable disease in humans. In our mouse model, we did not determine the survival time of the animals. Thus, a correlation between the grade of tumor growth impairment by meldonium with the survival was not possible based on the previous data.

Possible reasons for the variability in the response to meldonium in humans might be (i) heterogeneity of the tumor itself, (ii) differences in tumor treatment, but also (iii) a lack of compliance with the suggested treatment regime, or (iv) the late stage of GBM when the treatment was initiated. All three patients started their intake of meldonium in a highly progressive tumor state of the second relapse.

All three patients were male, had a similar age at diagnosis (66, 62, and 64 years). All tumors were IDH wildtype, but with either methylated (#1) or non-methylated (#2, #3) MGMT promoter status. All three patients received temozolomide (TMZ) according to the Stupp regimen [2] as first-line therapy after surgical resection of the primary tumor.

A difference in the therapy regime of patient #1 in comparison to the others is the lack of receiving any targeted therapy. Furthermore, meldonium administration in patient #1 was started in addition to TTF therapy, but TTF was discontinued three months later due to side effects (sleep disorders). Therefore, TTF application is unlikely to be responsible for the arrest in tumor progression in patient #1. Tumor progression occurred under TMZ alone or in combination with TTF, and the halt of tumor growth was achieved only after the addition of meldonium.

Several clinical studies have found a correlation between the prognosis and MGMT promoter methylation in patients treated with alkylating agents such as TMZ [17, 18]. Patient #1, in whom additional meldonium intake arrested tumor growth, had a methylated MGMT. Whether or not a methylated MGMT promoter was just a coincidental finding or in fact, is associated with response to meldonium cannot be judged at this point. In-depth analysis of the molecular profile will be needed to address the epigenetic and other molecular mechanisms.

Regarding possible reasons for the different efficacy of meldonium in our patients, it was recently published that GBM are heterogeneous in their fatty acid metabolism [19]. Thus, the more the tumor cells rely on FAO as an important energy source, the better response one might expect when interfering with this metabolic pathway. FAO supports GBM progression by providing energy and metabolic adaptability [6, 9]. Meldonium blocks OCTN2 and CPT1 and inhibits carnitine synthesis, leading to an overall reduction in FAO [13, 14]. As a result, tumor growth might be restricted. Moreover, carnitine is cell-protective by scavenging free radicals, stabilising membranes, and promoting antioxidant defences and anti-apoptotic pathways [20–22]. Thus, meldonium’s effects may rely on both FAO-dependent and independent mechanisms. While no other cancer studies on meldonium exist, FAO has been implicated in various cancers, such as breast [23–25], endometrial [26], and prostate [27], as well as non-small lung cancer [28]. In our case series, a differential efficacy of meldonium was observed. This might be based on a heterogeneity in GBM fatty acid metabolism [19]. Interestingly, modulation of fatty acid metabolism sensitizes recurrent GBM cells to temozolomide [19]. It is, therefore, conceivable that only a combined use of meldonium and temozolomide results in patient response. The metabolic profile of GBM and expression of glycolytic and mitochondrial FAO enzymes might be helpful in a subsequent clinical study to find an association with the potential response to meldonium.

Of note, only the patient (#1) who responded to meldonium application fully complied with our prescribed protocol for taking meldonium and the associated documentation procedure.

The patients presented were treated with various strategies as depicted in Table 1. Until now, patient #1 has survived more than five years since primary diagnosis and thus belongs to the rare long-term survivors. The five-year overall survival rate for GBM is only 6.9% [1]. Stupp et al. report that the median survival is 14.6 months for patients treated with radiochemotherapy and 12.1 months with radiotherapy alone [2]. There are no comparable studies on survival after the second recurrence. Only a survival time of 11.4 months after surgery of the first recurrence has been described [29]. There are also no data on the survival after growth arrest following permanent tumor progression, which overall makes a comparison with our cases difficult. Patient #1 and #3 had TTF in their treatment history. In recurrent GBM, the median survival of TTF patients was 6.6 compared to 6.0 months in the control group [30]. With regard to these survival data, meldonium-treated patient #1 had no progression of the second relapse for more than 24 months. However, all patients received temozolomide, combined with meldonium for patients #1 and #2, or only until the first relapse in patient #3. Studies indicate a link between prognosis and MGMT promoter methylation in patients treated with temozolomide [17, 18, 31]. Patient #1 had a methylated MGMT and experienced halted tumor growth. The relationship between a methylated MGMT promoter and meldonium response remains unassessed currently. Patients who did not respond to meldonium had unmethylated MGMT and an overall survival of 22 months (#2) and 26 months (#3), also exceeding GBM median survival.

Research indicates that TTF disrupts glycolysis through PKM2 inhibition [32, 33], thus TTF and meldonium together inhibit ATP production from both glycolysis and FAO, possibly accounting for the efficacy in patient #1. In contrast, anti-angiogenic therapy with bevacizumab enhances glycolytic enzyme expression and glucose metabolism in GBM [34, 35], potentially explaining the differential responses to meldonium. Patients #2 and #3 received anti-angiogenic therapy but did not respond to meldonium. All patients discussed underwent temozolomide therapy before starting additional therapies. It was observed that FAO activation contributes to temozolomide resistance in GBM [36]. Etomoxir, an FAO inhibitor, enhances the efficacy of temozolomide [19, 37]. Thus, the metabolic state might play a critical role in temozolomide efficacy, and FAO inhibition by meldonium may help combat drug resistance in GBM.

Certainly, this report has numerous limitations. Despite reporting outcomes in more than one patient, this remains a case report, lacking the ability to perform statistical analyses. We included patients with very different therapeutic backgrounds. Further, the missing of detailed pathologic workup of the tumor lesion seen in the MRI and the missing data regarding the molecular tumor pattern impede the proof of a therapeutic effect of meldonium. Based on this report clinical studies should follow e.g., a phase 2 clinical trial for patients with GBM that compares the existing standard therapy, including radiochemotherapy and optional TTF, with and without meldonium on top.

In conclusion, our data indicate a potential for meldonium to improve GBM treatment and emphasize the importance of conducting a clinical trial to systematically evaluate the effects of meldonium as a potential add-on therapy. A detailed molecular profiling should then be included to detect potential subgroups of patients who respond to meldonium.

## Supplementary information

Supplementary Figure S1

Supplementary Figure S2

Supplementary Figure legends

## Data Availability

The data that support the findings of this study are available on request from the corresponding author [SBM]. The data are not publicly available due to privacy and ethical restrictions, they contain information that could compromise the privacy of research participants.
