# Supplementary figures and images for: Case report of three patients with end-stage recurrent glioblastoma treated with meldonium
Source: BJC Rep. 2025 Apr 28;3:29. doi: 10.1038/s44276-025-00124-7 (PMC12037855; doi:10.1038/s44276-025-00124-7)

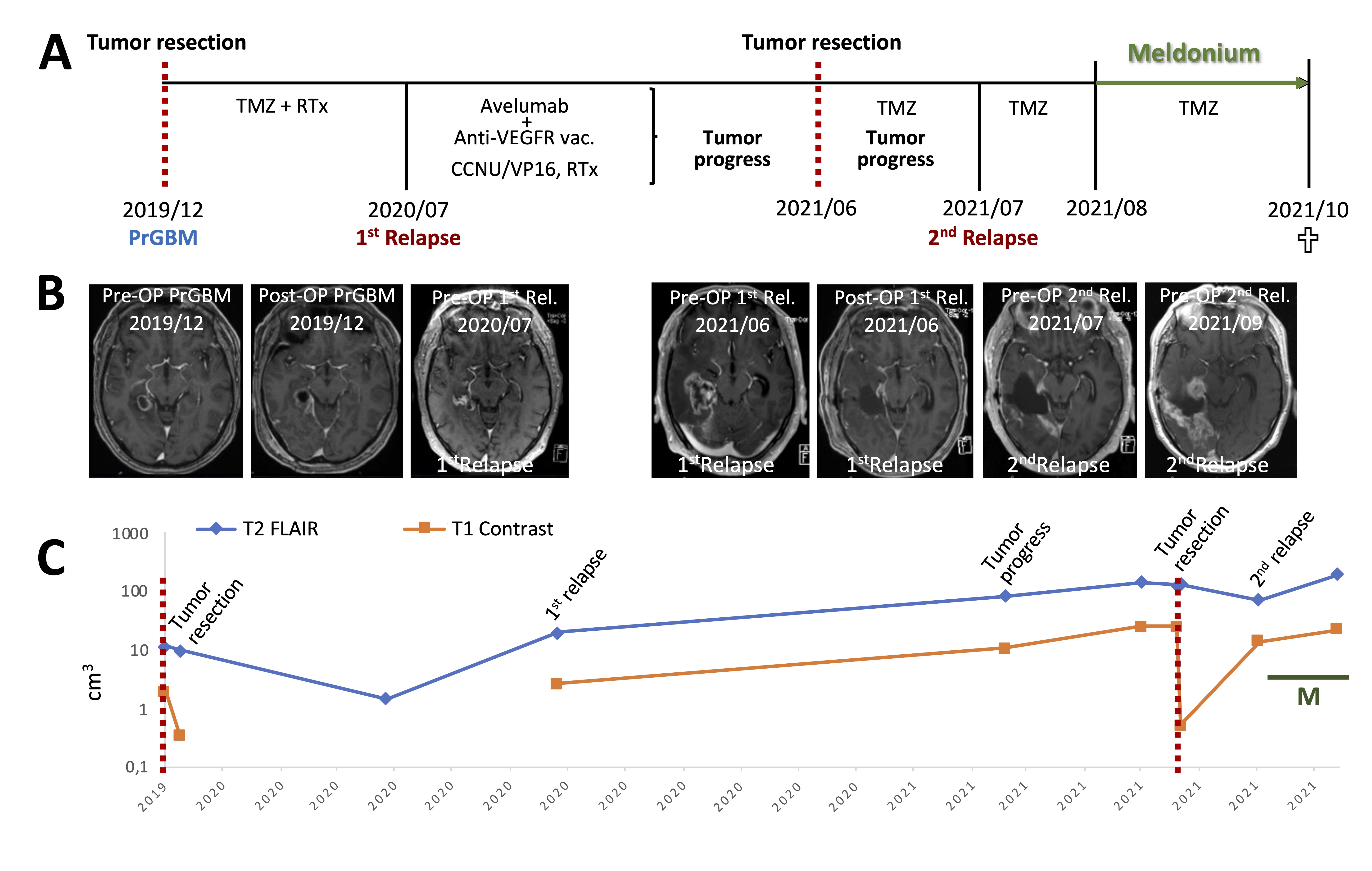

Supplement: Supplementary file 1 — Supplementary Figure S1 [file 44276_2025_124_MOESM1_ESM.jpg]
