# Supplementary material for: Case report of three patients with end-stage recurrent glioblastoma treated with meldonium
Source: BJC Rep. 2025 Apr 28;3:29. doi: 10.1038/s44276-025-00124-7 (PMC12037855; doi:10.1038/s44276-025-00124-7)
Supplement: Supplementary file 3 — Supplementary Figure legends [file 44276_2025_124_MOESM3_ESM.docx]

**Supplementary Figure S1.** Summary of GBM treatment and response from patient #2. (A) Clinical course and therapies over time. (B) Preoperative and postoperative axial sections of a T1 w MRI after gadolinium injection. (C) Tumor volumetry over the time. RTx, radiotherapy; TMZ, Temozolomide; TTF, Tumor Treating Fields; M, application of meldonium.

**Supplementary Figure S2.** Summary of GBM treatment and response from patient #3. (A) Clinical course and therapies over time. (B) Preoperative and postoperative axial sections of a T1 w MRI after gadolinium injection. (C) Tumor volumetry over the time. CCNU, Lomustine; RTx, radiotherapy; TMZ, Temozolomide; TTF, Tumor Treating Fields; M, application of meldonium.
