# Supplementary material for: Case report of three patients with end-stage recurrent glioblastoma treated with meldonium
Source: BJC Rep. 2025 Apr 28;3:29. doi: 10.1038/s44276-025-00124-7 (PMC12037855; doi:10.1038/s44276-025-00124-7)
Supplement: Supplementary file 2 — Supplementary Figure S2 [file 44276_2025_124_MOESM2_ESM.pdf]

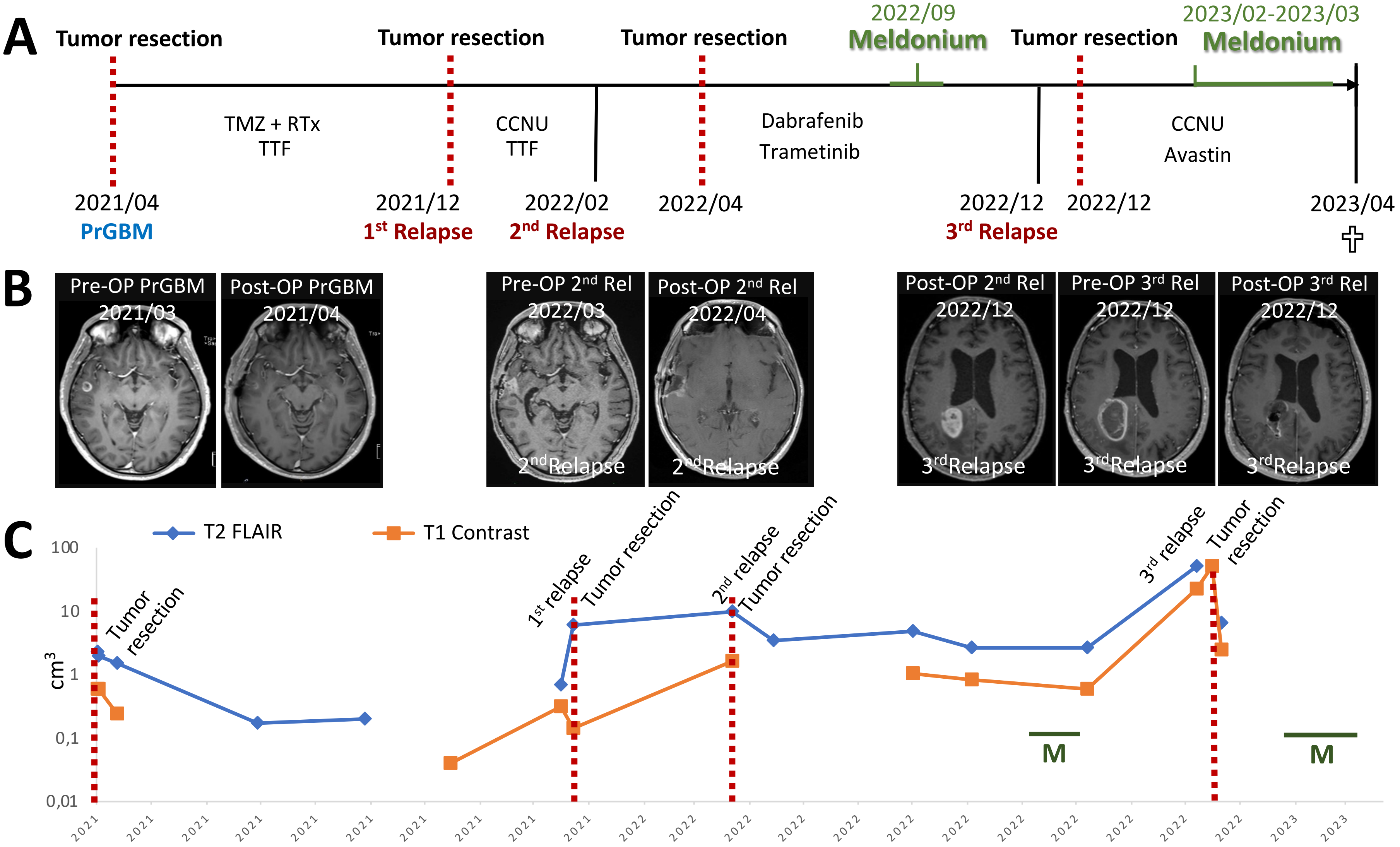
A
Tumor resection
Tumor resection
Tumor resection
2022/09
Meldonium
Tumor resection
2023/02-2023/03
Meldonium
TMZ + RTx
TTF
CCNU
TTF
Dabrafenib
Trametinib
CCNU
Avastin
2021/04
PrGBM
2021/12
1st Relapse
2022/02
2nd Relapse
2022/04
2022/12
3rd Relapse
2022/12
2023/04
B
Pre-OP PrGBM
2021/03
Post-OP PrGBM
2021/04
Pre-OP 2nd Rel
2022/03
2nd Relapse
Post-OP 2nd Rel
2022/04
2nd Relapse
Post-OP 2nd Rel
2022/12
3rd Relapse
Pre-OP 3rd Rel
2022/12
3rd Relapse
Post-OP 3rd Rel
2022/12
3rd Relapse
C
T2 FLAIR
T1 Contrast
cm3
100
10
1
0,1
0,01
Tumor resection
1st relapse
Tumor resection
2nd relapse
Tumor resection
3rd relapse
Tumor resection
M
M
2021
2022
2023
